# Supplementary material for: Geometrical Stabilities and Electronic Structures of Ru3 Clusters on Rutile TiO2 for Green Hydrogen Production
Source: Nanomaterials (Basel). 2024 Feb 21;14(5):396. doi: 10.3390/nano14050396 (PMC10934880; doi:10.3390/nano14050396)
Supplement: Supplementary file 1 [file nanomaterials-14-00396-s001.zip › nanomaterials-2853720-supplementary.pdf]

Geometrical Stabilities and Electronic Structures of Ru_3 Clusters on Rutile TiO_2 for Green Hydrogen Production

Moteb Alotaibi

Department of Physics, College of Science and Humanities in Al-Kharj, Prince Sattam Bin Abdulaziz
University, Al-Kharj 11942, Saudi Arabia; mot.alotaibi@psau.edu.sa

1. Pristine rutile TiO_2 (110) surface

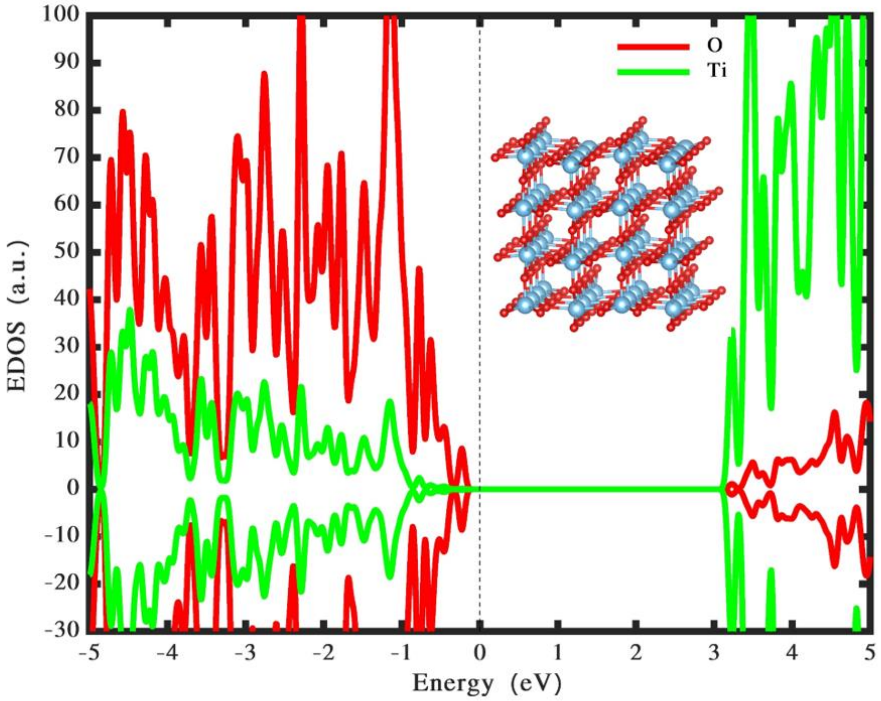

Figure S1 Density of states of pristine rutile TiO_2 (110). The green and red curves show the electronic density of states on titanium and oxygen atoms, respectively. The black vertical dashed line shows the Fermi energy level. Reproduced from our previous calculations [1].

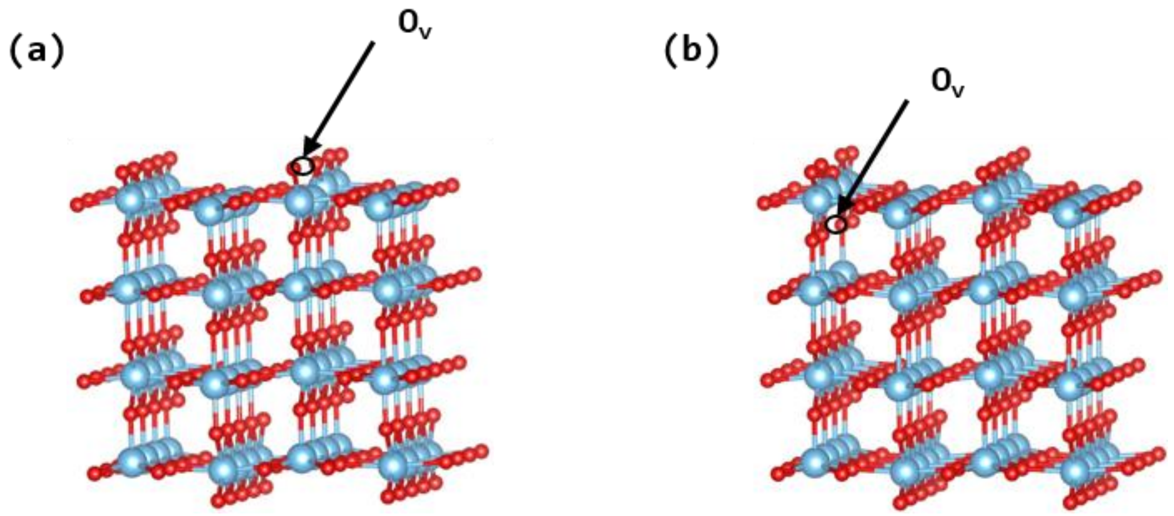

Figure S2 Oxygen vacancy formation at (a) surface and (b) subsurface locations of TiO_2 rutile (110). The black circles represent the oxygen vacancy position. Reproduced from our previous calculations [1].

2. Ru_3@reduced TiO_2

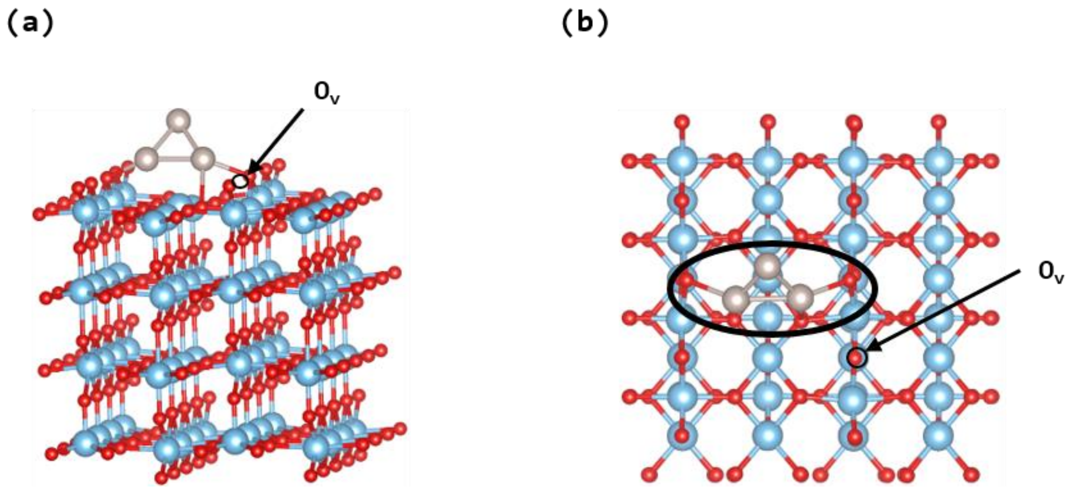

Figure S3 Oxygen vacancy formation at (a) top view and (b) lateral view of the most stable Ru_3@TiO_2 rutile (110). The black circles show the oxygen vacancy position.

Table S1 Comparisons of formation energies of oxygen vacancy for structures shown in Figure S2 and Figure S3.

Structure	(a) in Figure S2	(b) in Figure S2	(a) in Figure S3
**Formation energy of oxygen vacancy (eV)**	4.07	4.65	4.45

References

[1] M. Alotaibi, Q. Wu, and C. Lambert, “Computational Studies of Ag 5 Atomic Quantum Clusters Deposited on Anatase and Rutile TiO 2 Surfaces,” *Appl Surf Sci*, p. 156054, 2022, doi: 10.1016/j.apsusc.2022.156054.
